# The quality of guidelines for treatment of carotid artery disease: a critical appraisal using the AGREE II instrument

**DOI:** 10.1590/1677-5449.202200321

**Published:** 2022-11-25

**Authors:** Stefany Gimenes Baptista Coutinho, Joelma Cavalcante Ricardo, Alexandre Inacio Moreira Coutinho, Leonardo Pessoa Cavalcante

**Affiliations:** 1 Universidade Federal do Amazonas - UFAM, Manaus, AM, Brasil.; 2 Marinha do Brasil, Policlínica Naval de Manaus, Manaus, AM, Brasil.; 3 Fundação Centro de Controle de Oncologia do Estado do Amazonas, Manaus, AM, Brasil.; 4 Sociedade Brasileira de Angiologia e Cirurgia Vascular, Regional Amazonas, Manaus, AM, Brasil.; 5 Universidade Federal do Amazonas - UFAM, Hospital Universitário Getúlio Vargas, Manaus, AM, Brasil.

**Keywords:** practice guideline, carotid artery diseases, atherosclerosis, carotid stenosis

## Abstract

Clinical Practice Guidelines (CPG) are structured recommendations based on systematic reviews of the available evidence and are useful tools to support clinical decision-making. However, studies have raised concerns about the methodological and scientific quality of several CPG, which can affect their application in clinical practice. The objective of this study was to perform a systematic appraisal of the methodological quality of carotid atherosclerotic disease clinical guidelines, published from 2000 to 2019, using the AGREE II instrument (Appraisal of Guidelines for Research and Evaluation Instrument II). The appraisers independently assessed the quality of the CPG included in the study for each of the 6 domains of the AGREE II tool. The CPG were rated as high, moderate, or low quality using a points scale. A total of 9 CPGs were selected for appraisal. Except for domain 2 (kappa=0.715), excellent agreement was observed between the appraisers (kappa>0.75). Five of the CPGs were rated as high overall methodological quality rating, 5 were rated as moderate overall methodological quality, and 2 were rated low overall methodological quality. The authors conclude that: (1) appraisal of carotid atherosclerotic disease clinical guidelines using the AGREE II instrument is feasible, with a high degree of agreement among appraisers; and (2) that most CPGs on the management of atherosclerotic carotid disease have high methodological quality.

## INTRODUCTION

Clinical practice guidelines (CPGs) are structured recommendations developed to support healthcare professionals in decision-making for individual patients in specific circumstances, based on a systematic review of the available evidence and on the risks and benefits of the available therapeutic options.^1^^,^^2^ Healthcare professionals, managers, and healthcare financers see CPGs as tools that can close the gap between healthcare practice and the scientific evidence yielded by clinical trials conducted in controlled settings.^3^^,^^4^ Therefore, CPGs based on the best available scientific evidence provide a basis for clinical decision-making taking into account each patient’s individual clinical characteristics and also support healthcare managers tasked with regulating healthcare systems.^5^ However, several different studies have identified CPGs of low to moderate scientific methodological quality, raising concerns among the healthcare professionals who apply them in their decision-making.^6^^,^^7^

Several tools for assessment of CPGs quality have been developed.^8^^,^^9^ The Appraisal of Guidelines for Research and Evaluation Instrument (AGREE)^10^ and its second version (AGREE II), published in 2009,^11^ have gained recognition and have been validated and are widely used in several different languages,^12^^,^^13^^,^^14^ including Portuguese.^14^ The AGREE II^11^ instrument has been used in Brazil with increasing frequency over recent years and is used by the country’s Ministry of Health as part of its process of CPGs development,^15^ for assessment of CPGs for treatment of non-transmissible diseases^16^ and to support CPGs development by specialist physicians from a variety of specialties.^17^^,^^18^

The AGREE II tool^11^ is designed for: (1) healthcare professionals, who can use it to assess a guideline before adopting its recommendations in clinical practice; (2) for guideline writers, so that they can employ a structured and rigorous development methodology; (3) for those responsible for managing healthcare policies, to enable them to decide which CPGs can be used to support healthcare policy decision-making; and (4) for educators, to help them improve critical assessment skills and emphasize which competencies are essential to CPGs development to ensure that they can be used to support clinical decision-making. A total of 33 official translations of the AGREE^10^ and AGREE II instruments^11^ are available for use by the international community.^11^

Extracardiac vascular diseases have a high prevalence in the global population over the age of 60 years. Extracranial obstructive carotid artery disease (CAD) is a condition in which the great majority of surgical interventions are conducted in individuals who are entirely asymptomatic.^19^ Considering that this condition has zero impact on the lives of asymptomatic individuals and that there are no interventions, whether surgical or drug-based, that involve zero risk (or cost), a systematic review of the quality of the CPGs that guide treatment of this disease is justified, with the objective of supporting both physicians in their decision-making and healthcare system managers and financers who need to assess whether available resources are being employed rationally.

Anatomically, CAD is characterized by stenosis or occlusion of the carotid artery that, in the majority of cases, is secondary to atherosclerotic processes that primarily affect the carotid bifurcation, and it is responsible for approximately 20% of cases of ischemic stroke (IS).^20^ Treatment of CAD consists of drug-based clinical treatment, with rigorous clinical control of associated diseases (systemic arterial hypertension, diabetes mellitus, dyslipidemia, and smoking) and of surgical interventions in selected cases (carotid endarterectomy or angioplasty with stenting).^19^^,^^20^ For symptomatic patients, there is consensus on surgical treatment for secondary prevention for a repeat IS; whereas surgical treatment of stenosis remains controversial in cases in which patients diagnosed with CAD are asymptomatic.^20^

This study is a systematic appraisal of the methodological quality of CPGs that cover treatment of CAD, using the AGREE II tool.

## METHODS

Searches were run on two electronic bibliographic databases (PubMed/MEDLINE and SciELO) and on Google Scholar. The following keywords were used: “carotid artery disease”, “atherosclerotic”, and “practice guideline”. Publications classified as CPG or as consensus statements covering treatment of CAD and published from 2000 to 2019 were included. Publications in languages other than English, Portuguese, or Spanish were excluded. Two appraisers conducted the initial screening of studies for inclusion and selection of the CPGs for analysis by reading titles and abstracts.

Three appraisers (SGBC, JCR, and LPC) independently assessed the CPGs included in the review scoring them from 1 (low quality) to 7 (high quality), for each of the 23 items across the six domains of the AGREE II tool: 1) scope and purpose; 2) stakeholder involvement; 3) rigor of development; 4) clarity of presentation; 5 applicability; and 6) editorial independence; (Table 1). The following formula^12^ was used to generate a weighted score (as a percentage) for each domain: (score awarded - minimum possible score)/(maximum possible score - minimum possible score) × 100. All assessments and ratings were performed as described in the AGREE II user’s manual, available on the AGREE Research Trust website.^11^

**Table 1:** Items and domains of the AGREE II instrument (Appraisal of Guidelines for Research and Evaluation Instrument II)*.

**Item**	**Content**	**Domain**
1	The overall objective(s) of the guideline is (are) specifically described.	**Scope and purpose**
2	The health question(s) covered by the guideline is (are) specifically described.	
3	The population (patients, public, etc.) to whom the guideline is meant to apply is specifically described.	
4	The guideline development group includes individuals from all the relevant professional groups.	**Stakeholder involvement**
5	The views and preferences of the target population (patients, public, etc.) have been sought.	
6	The target users of the guideline are clearly defined.	
7	Systematic methods were used to search for evidence.	**Rigor of development**
8	The criteria for selecting the evidence are clearly described.	
9	The strengths and limitations of the body of evidence are clearly described.	
10	The methods for formulating the recommendations are clearly described.	
11	The health benefits, side effects, and risks have been considered in formulating the recommendations.	
12	There is an explicit link between the recommendations and the supporting evidence.	
13	The guideline has been externally reviewed by experts	
14	A procedure for updating the guideline is provided.	
15	The recommendations are specific and unambiguous.	**Clarity of presentation**
16	The different options for management of the condition or health issue are clearly presented.	
17	Key recommendations are easily identifiable	
18	The guideline describes facilitators and barriers to its application.	**Applicability**
19	The guideline provides advice and/or tools on how the recommendations can be put into practice.	
20	The potential resource implications of applying the recommendations have been considered.	
21	The guideline presents monitoring and/ or auditing criteria.	
22	The views of the funding body have not influenced the content of the guideline.	**Editorial independence**
23	Competing interests of guideline development group members have been recorded and addressed.	

* Extracted from: AGREE Next Steps Consortium.^11^

The degree of agreement between appraisers was calculated using the Fleiss Kappa coefficient of agreement.^21^ As recommended by Landis and Koch,^22^ Fleiss Kappa values ≥ 0.75 were defined as representation excellent agreement between appraisers. Domains for which the Fleiss Kappa value was < 0.75 would be discussed and reviewed in a meeting of all three appraisers, regardless of individual scores.

**Figure 1:**
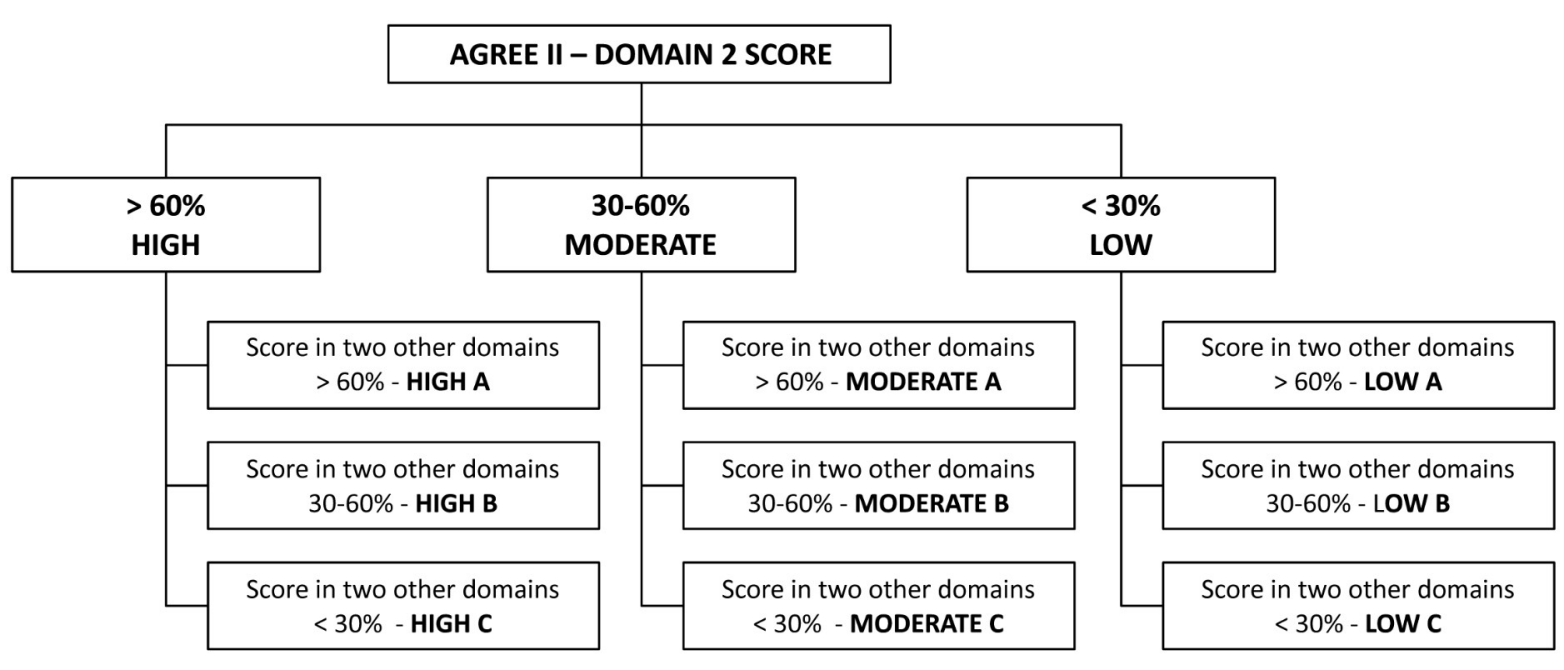
Metric used for methodological quality rating of each guideline (extracted from Molino et al.^16^). AGREE II: Appraisal of Guidelines for Research and Evaluation Instrument II.

The overall methodological quality of each CPG was rated using the metric proposed by Molino et al.,^16^ which prioritizes domain 3, since this is domain assessing the CPG’s methodological rigor of development. The result for domain 3 was therefore used to categorize the CPG as “high”, “moderate”, or “low” quality. They were then subclassified as A, B, or C by analysis of the next two best performing domains according to the AGREE II tool (Figure 1).

## RESULTS

Nine CPGs^23^^-^31 were identified and listed by year of publication (Table 2). Eight CPGs were published in English, found in the PubMed/MEDLINE search, and one was written in Portuguese, identified by Google Scholar. The majority of the CPGs identified were published since 2010 (66%), i.e., in the second half of the period stipulated for the search.

**Table 2:** General information on the nine clinical guidelines.

**NO.**	**Country**	**Title**	**Year of publication**
1	International (European)	2017 ESC Guidelines on the diagnosis and treatment of peripheral arterial diseases, in collaboration with the European Society for Vascular Surgery.	2017^23^
2	International (European)	Management of atherosclerotic carotid and vertebral artery disease: 2017 clinical practice guidelines of the European Society for Vascular Surgery.	2017^24^
3	Brazil	Doença carotídea extracraniana. Diagnóstico e tratamento.	2015^25^
4	United States	ASA/ACCF/AHA/AANN/AANS/ACR/ASNR/CNS/SAIP/SCAI/SIR/SNIS/SVM/SVS. Guideline on the Management of Patients With Extracranial Carotid and Vertebral Artery Disease: Executive Summary.	2011^26^
5	United States	Updated Society for Vascular Surgery guidelines for management of extracranial carotid disease.	2011^27^
6	United Kingdom	Carotid artery stent placement for asymptomatic extracranial carotid stenosis.	2011^28^
7	United States	Management of carotid atherosclerotic disease: Clinical practice guidelines of the Society for Vascular Surgery.	2008^29^
8	United States	Primary prevention of ischemic stroke.	2006^30^
9	United Kingdom	Guidelines on the management of secondary prophylaxis of vascular events in stable patients in primary care.	2004^31^

The initial scores for each domain and the initial overall assessment for the nine CPGs assessed are shown in Table 3. Except for domain 2 (stakeholder involvement), there was excellent agreement between appraisers. The three appraisers therefore held a meeting to discuss and possibly revise the scores awarded to the CPG for domain 2. After this discussion meeting, each appraiser was completely free to revise (or not) their original scores, and then the coefficient of agreement was recalculated. The final result was then rated as excellent inter-appraiser agreement (excellent or total agreement) (Table 4).

**Table 3:** Initial scores in the six domains for the nine clinical guidelines assessed with the AGREE II tool (Appraisal of Guidelines for Research and Evaluation Instrument II).

**CPG (year)**	**Domain 1** Scope and purpose*	**Domain 2** Stakeholder involvement*	**Domain 3** Rigor of development*	**Domain 4** Clarity of presentation*	**Domain 5** Applicability*	**Domain 6** Editorial independence*
**1 (2017)**	100.00	80.00	98.61	100.00	84.72	100.00
**2 (2017)**	100.00	100.00	91.60	100.00	91.60	100.00
**3 (2015)**	72.22	3.70	16.66	78.18	19.44	19.44
**4 (2011)**	98.14	96.29	96.52	100.00	80.00	80.00
**5 (2011)**	77.77	88.88	77.77	100.00	68.05	72.22
**6 (2011)**	100.00	96.29	92.36	98.14	72.22	88.88
**7 (2008)**	100.00	77.77	83.33	100.00	58.33	94.44
**8 (2006)**	83.33	75.92	75.69	92.59	66.66	91.66
**9 (2004)**	98.14	79.62	56.94	96.29	58.33	69.44
Fleiss Kappa**	0.962	0.715	0.793	0.928	0.889	0.803

CPG: clinical practice guideline.

* Scores as percentages;

** Fleiss Kappa coefficient of agreement.

**Table 4:** Final scores (after meeting of appraisers) for the six domains and overall methodological quality rating for the nine clinical guidelines, using the AGREE II tool (Appraisal of Guidelines for Research and Evaluation Instrument II).

**CPG (year)**	**Domain 1** Scope and purpose*	**Domain 2** Stakeholder involvement*	**Domain 3** Rigor of development*	**Domain 4** Clarity of presentation*	**Domain 5** Applicability*	**Domain 6** Editorial independence*	Overall methodological quality rating**
**1 (2017)**	100.00	80.00	98.61	100.00	84.72	100.00	HIGH - A
**2 (2017)**	100.00	100.00	91.60	100.00	91.60	100.00	HIGH - A
**3 (2015)**	72.22	1.80	16.66	78.18	19.44	19.44	LOW - A
**4 (2011)**	98.14	96.29	96.52	100.00	80.00	80.00	HIGH - A
**5 (2011)**	77.77	88.88	77.77	100.00	68.05	72.22	MODERATE - A
**6 (2011)**	100.00	96.29	92.36	98.14	72.22	88.88	HIGH - A
**7 (2008)**	100.00	77.77	83.33	100.00	58.33	94.44	HIGH - A
**8 (2006)**	83.33	57.40	75.69	92.59	66.66	91.66	MODERATE - A
**9 (2004)**	98.14	74.10	56.94	96.29	58.33	69.44	LOW - A
kappa***	0.962	0.813	0.793	0.928	0.889	0.803	-

CPG: clinical practice guideline.

* Scores as percentages;

** Overall methodological quality rating using metric proposed by Molino et al.^16^;

*** Fleiss Kappa coefficient of agreement.

According to the overall methodological quality assessment for the CPGs using the metric proposed by Molino et al.,^16^ five guidelines were rated high quality (HIGH A), which corresponds to 55% of the CPGs assessed; two guidelines were rated moderate quality (MODERATE A); and two guidelines were rated low quality (LOW A) (Table 4).

## DISCUSSION

With regard to the overall methodological quality ratings for these CPGs, if we only consider those published in English and available on PubMed/MEDLINE, 62.5% were rated high quality (all HIGH A). With regard to the negative assessment of the overall methodological quality of the only CPG available in Portuguese, as was also found in quality assessments of CPGs on cardiac diseases^32^ and other chronic diseases,^16^^,^^33^ there is ample room for improving methodological quality using instruments such as the AGREE II tool during the design phase. Equally important is the evident need for those reading CPGs and treating physicians to also employ easy-to-use tools such as the AGREE II during their critical reading of the CPGs that they use to support their patient management decisions.^32^

Hoffmann-Eßer et al.^34^ conducted an online survey of 376 researchers, finding that the domains that had greatest impact on the overall methodological quality rating of a CPG are domains 3 (rigor of development) and six (editorial independence). In our study, the two CPG that were rated low quality also had the worst scores in these two domains. These findings underscore the importance of domain 3, which has the highest number of items (eight), and of domain 6 (despite only having two items).

It is presumed that the year of publication of the ninth CPG (2004), 1 year after the first version of the AGREE instrument was published,^10^ is a relevant factor in its low overall methodological quality, since the AGREE tool was still not widely used then. Although the third CPG is more recent, its design may not have taken the AGREE II tool^11^ or other similar instruments^8^^,^^9^ into consideration.

One drawback with regard to use of the AGREE II tool is the wide range of variation (1 to 7) in the scale appraisers use to rate each of the 23 items, which can lead to some degree of subjectivity on the part of each appraiser.^32^ However, the high degree of inter-appraiser agreement we observed shows that the AGREE II instrument can overcome this possible bias. Even in the only domain in which discussion and re-rating were conducted (domain 2), the degree of initial agreement was already close to the cutoff for excellent (an initial Fleiss Kappa value of 0.715). This is consistent with studies that have confirmed the validity of the AGREE II as a tool for CPGs assessment in a variety of medical specialties, even when there is a degree of heterogeneity among appraisers.^35^

Certain criticisms of the applicability of the AGREE II instrument to CPGs for surgical diseases were made in a recent protocol proposal,^36^ which highlighted the difficulties of dealing with cost-effectiveness due to several different reasons: because it is an item rarely covered in the surgical literature, because of the variability of surgical experience in different countries, because of the need for application of the instrument in different healthcare settings, and because of the need for common comparators to cover the details of more complex interventions. This protocol therefore proposed an extension of the AGREE instrument for evaluation of CPGs for surgical diseases, with inclusion of a checklist of specific items related to their specific characteristics. The objective of developing this extension would therefore be to expand the instrument’s applicability and increase its value for CPGs for clinical practice in surgery.

One limitation of the present study was the participation of only a specific subset of appraisers. The three appraisers were physicians, vascular surgery specialists, whose main activity is patient care, with no participation by other appraisers, such as management, educators, and/or CPG writers. There was also no participation by treating physicians from other specialties who take part in the decision-making process for patients with CAD (p. ex., neurologists and cardiologists). Another drawback is that the AGREE II instrument was developed to assess the methodological quality of CPGs development and is not appropriate for analysis specifically of the merit of their content, as already pointed out by other authors who have used the instrument to assess the quality of CPGs focused on other vascular diseases.^37^

## CONCLUSIONS

On the basis of our findings, the following can be concluded: (1) it proved feasible to employ the AGREE II to assess CPGs on treatment of CAD with a high degree of agreement between appraisers; and (2) the majority of CPGs on treatment of CAD published in English are of high overall methodological quality.

## References

[1] Field MJ, Lohr KN (1990). Clinical practice guidelines: directions for a new program.

[2] Graham R, Mancher M, Wolman DM, Greenfield S, Steinberg E (2011). Clinical practice guidelines we can trust.

[3] Woolf SH, Grol R, Hutchinson A, Eccles M, Grimshaw J (1999). Clinical guidelines: potential benefits, limitations, and harms of clinical guidelines. BMJ.

[4] Grol R (2001). Successes and failures in the implementation of evidence-based guidelines for clinical practice. Med Care.

[5] Reis ECD, Passos SRL, Santos MABD (2018). Quality assessment of clinical guidelines for the treatment of obesity in adults: application of the AGREE II instrument. Cad Saúde Pública.

[6] Al-Ansary LA, Tricco AC, Adi Y (2013). A systematic review of recent clinical practice guidelines on the diagnosis, assessment and management of hypertension. PLoS One.

[7] Kung J, Miller RR, Mackowiak PA (2012). Failure of clinical practice guidelines to meet institute of medicine standards: two more decades of little, if any, progress. Arch Intern Med.

[8] Vlayen J, Aertgeerts B, Hannes K, Sermeus W, Ramaekers D (2005). A systematic review of appraisal tools for clinical practice guidelines: multiple similarities and one common deficit. Int J Qual Health Care.

[9] Siering U, Eikermann M, Hausner E, Hoffmann-Eßer W, Neugebauer EA (2013). Appraisal tools for clinical practice guidelines: a systematic review. PLoS One.

[10] AGREE Collaboration (2003). Development and validation of an international appraisal instrument for assessing the quality of clinical practice guidelines: the AGREE project. Qual Saf Health Care.

[11] AGREE Next Steps Consortium (2009). AGREE Next Steps Consortium.

[12] Alonso-Coello P, Irfan A, Solà I (2010). The quality of clinical practice guidelines over the last two decades: a systematic review of guideline appraisal studies. Qual Saf Health Care.

[13] Legido-Quigley H, Panteli D, Brusamento S (2012). Clinical guidelines in the European Union: mapping the regulatory basis, development, quality control, implementation and evaluation across member states. Health Policy.

[14] Khan GS, Stein AT (2014). Adaptação transcultural do instrumento Appraisal of Guidelines for Research & Evaluation II (AGREE II) para avaliação de diretrizes clínicas. Cad Saúde Pública.

[15] Ronsoni RM, Pereira CC, Stein AT, Osanai MH, Machado CJ (2015). Avaliação de oito Protocolos Clínicos e Diretrizes Terapêuticas (PCDT) do Ministério da Saúde por meio do instrumento AGREE II: um estudo piloto. Cad Saúde Pública.

[16] Molino CG, Romano-Lieber NS, Ribeiro E, Melo DO (2016). Non-communicable disease clinical practice guidelines in Brazil: a systematic assessment of methodological quality and transparency. PLoS One.

[17] Cotrim HP, Parise ER, Figueiredo-Mendes C, Galizzi-Filho J, Porta G, Oliveira CP (2016). Nonalcoholic fatty liver disease Brazilian Society of Hepatology Consensus. Arq Gastroenterol.

[18] Westphal GA, Garcia VD, Souza RL (2016). Guidelines for the assessment and acceptance of potential brain-dead organ donors. Rev Bras Ter Intensiva.

[19] Abbott AL (2009). Medical (nonsurgical) intervention alone is now best for prevention of stroke associated with asymptomatic severe carotid stenosis: results of a systematic review and analysis. Stroke.

[20] Sitrângulo CJ, Silva ES (2018). Carotid atherosclerotic disease. J Vasc Bras.

[21] Fleiss JL, Levin B, Paik MC (2003). Statistical method for rates and proportions.

[22] Landis JR, Koch GG (1977). The measurement of observer agreement for categorical data. Biometrics.

[23] Aboyans V, Ricco JB, Bartelink MEL (2018). 2017 ESC Guidelines on the Diagnosis and Treatment of Peripheral Arterial Diseases, in collaboration with the European Society for Vascular Surgery (ESVS): document covering atherosclerotic disease of extracranial carotid and vertebral, mesenteric, renal, upper and lower extremity arteries. Endorsed by: the European Stroke Organization (ESO) the Task Force for the Diagnosis and Treatment of Peripheral Arterial Diseases of the European Society of Cardiology (ESC) and of the European Society for Vascular Surgery (ESVS). Eur Heart J.

[24] Naylor AR, Ricco JB, Borst GJ (2018). Management of atherosclerotic carotid and vertebral artery disease: 2017 clinical practice guidelines of the European Society for Vascular Surgery (ESVS). Eur J Vasc Endovasc Surg.

[25] Presti C, Miranda FM, Silva JCCB (2015). Doença carotídea extracraniana - diagnóstico e tratamento.

[26] Brott TG, Halperin JL, Abbara S (2011). 2011ASA/ACCF/AHA/AANN/AANS/ACR/ASNR/CNS/SAIP/SCAI/SIR/SNIS/SVM/SVS guideline on the management of patients with extracranial carotid and vertebral artery disease: executive summary. Stroke.

[27] Ricotta JJ, Aburahma A, Ascher E, Eskandari M, Faries P, Lal BK (2011). Updated Society for Vascular Surgery guidelines for management of extracranial carotid disease. J Vasc Surg.

[28] National Institute for Health and Clinical Excellence (2011). Carotid artery stent placement for asymptomatic extracranial carotid stenosis.

[29] Hobson RW, Mackey WC, Ascher E (2008). Management of atherosclerotic carotid artery disease: clinical practice guidelines of the Society for Vascular Surgery. J Vasc Surg.

[30] Goldstein LB, Adams R, Alberts MJ (2006). Primary prevention of ischemic stroke: a guideline from the American Heart Association/American Stroke Association Stroke Council: cosponsored by the Atherosclerotic Peripheral Vascular Disease Interdisciplinary Working Group; Cardiovascular Nursing Council; Clinical Cardiology Council; Nutrition, Physical Activity, and Metabolism Council; and the Quality of Care and Outcomes Research Interdisciplinary Working Group: the American Academy of Neurology affirms the value of this guideline. Stroke.

[31] Betteridge DJ, Belch J, Brown MM (2004). Guidelines on the management of secondary prophylaxis of vascular events in stable patients in primary care. Int J Clin Pract.

[32] Sabharwal S, Patel V, Nijjer SS (2013). Guidelines in cardiac clinical practice: evaluation of their methodological quality using the AGREE II instrument. J R Soc Med.

[33] Shaneyfelt TM, Mayo-Smith MF, Rothwangl J (1999). Are guidelines following guidelines? The methodological quality of clinical practice guidelines in the peer-reviewed medical literature. JAMA.

[34] Hoffmann-Eßer W, Siering U, Neugebauer EAM, Brockhaus AC, McGauran N, Eikermann M (2018). Guideline appraisal with AGREE II: online survey of the potential influence of AGREE II items on overall assessment of guideline quality and recommendation for use. BMC Health Serv Res.

[35] Brouwers MC, Kho ME, Browman GP (2010). Development of the AGREE II, part 2: assessment of validity of items and tools to support application. CMAJ.

[36] Antoniou GA, Mavridis D, Tsokani S (2020). Protocol of an interdisciplinary consensus project aiming to develop an AGREE II extension for guidelines in surgery. BMJ Open.

[37] Zhang P, Lu Q, Li H (2019). The quality of guidelines for diabetic foot ulcers: A critical appraisal using the AGREE II instrument. PLoS One.

