# Supplementary material for: The quality of guidelines for treatment of carotid artery disease: a critical appraisal using the AGREE II instrument
Source: J Vasc Bras. 2022 Nov 25;21:e20220032. doi: 10.1590/1677-5449.202200321 (PMC9716357; doi:10.1590/1677-5449.202200321)
Supplement: Translation

# Translation: Qualidade metodológica das diretrizes de tratamento da doença arterial obstrutiva carotídea: uma avaliação sistemática com a utilização do instrumento AGREE II

**DOI:** 10.1590/1677-5449.202200322

**Authors:** Stefany Gimenes Baptista Coutinho, Joelma Cavalcante Ricardo, Alexandre Inacio Moreira Coutinho, Leonardo Pessoa Cavalcante

**Affiliations:** 1 Universidade Federal do Amazonas - UFAM, Manaus, AM, Brasil.; 2 Marinha do Brasil, Policlínica Naval de Manaus, Manaus, AM, Brasil.; 3 Fundação Centro de Controle de Oncologia do Estado do Amazonas, Manaus, AM, Brasil.; 4 Sociedade Brasileira de Angiologia e Cirurgia Vascular, Regional Amazonas, Manaus, AM, Brasil.; 5 Universidade Federal do Amazonas - UFAM, Hospital Universitário Getúlio Vargas, Manaus, AM, Brasil.

**Keywords:** guia de prática clínica, doenças das artérias carótidas, aterosclerose, estenose das carótidas

## Abstract

Diretrizes clínicas (DCs) são recomendações estruturadas baseadas na revisão sistemática da evidência disponível, sendo ferramentas úteis na tomada de decisões clínicas. Entretanto, estudos têm levantado preocupação quanto à qualidade metodológica e científica de várias DCs, que podem afetar sua aplicação na prática clínica. O objetivo do presente estudo foi fazer uma avaliação sistemática da qualidade metodológica das DCs que abordam o tratamento da doença arterial obstrutiva carotídea, publicadas entre 2000 e 2019, utilizando a ferramenta AGREE II (*Appraisal of Guidelines Research and Evaluation Instrument II*). Os pesquisadores avaliaram independentemente a qualidade das DCs incluídas no estudo em cada um dos seis domínios da ferramenta AGREE II. Por meio de um sistema de pontuação, as DCs foram classificadas em alta, moderada e baixa qualidade. Um total de nove DCs foram selecionadas. Exceto pelo domínio dois (Kappa = 0,715), houve concordância excelente entre os três avaliadores (Kappa > 0,75). Considerando-se a avaliação global da qualidade metodológica das DCs, cinco foram consideradas de alta qualidade (55%), duas foram consideradas de qualidade moderada e duas foram consideradas de baixa qualidade. Concluímos que (1) foi factível a utilização da AGREE II para a avaliação de DCs sobre o tratamento da doença arterial obstrutiva carotídea com alto grau de concordância inter-avaliadores; e que (2) a maioria das DCs disponíveis sobre o tratamento da doença arterial obstrutiva carotídea tem alta qualidade metodológica.

## INTRODUÇÃO

As diretrizes clínicas (DCs) são recomendações estruturadas desenvolvidas para auxiliar profissionais de saúde na tomada de decisão frente a pacientes individuais, em circunstâncias específicas, baseadas na revisão sistemática da evidência disponível e na avaliação de riscos e benefícios das alternativas terapêuticas disponíveis^1^^,^^2^. Os profissionais de saúde, bem como gestores e financiadores de saúde, enxergam as DCs como ferramentas capazes de reduzir o hiato existente entre a prática médica assistencial e a evidência científica oriunda de ensaios clínicos realizados em cenários controlados^3^^,^^4^. Portanto, as DCs baseadas na melhor evidência científica disponível servem para embasar a tomada de decisão clínica frente às particularidades de cada paciente, bem como para auxiliar gestores na regulação de sistemas de saúde^5^. Entretanto, vários estudos têm identificado DCs de qualidade científica metodológica baixa a moderada, o que levanta uma preocupação nos profissionais de saúde que as utilizam para tomar decisões^6^^,^^7^.

Algumas ferramentas têm sido desenvolvidas para a avaliação da qualidade de DCs^8^^,^^9^. Entre elas, se destacam a *Appraisal of Guidelines Research and Evaluation Instrument* (AGREE)^10^ e sua segunda versão (AGREE II), publicada em 2009^11^, amplamente utilizada e validada em vários idiomas^12^^,^^13^^,^^14^, inclusive na língua portuguesa^14^. A AGREE II^11^ tem sido utilizada no Brasil com maior frequência nos últimos anos, tendo sido aplicada no processo de elaboração de DCs elaboradas pelo Ministério da Saúde^15^, na avaliação de DCs do tratamento de doenças não infecciosas^16^ e no suporte da elaboração de DCs por médicos especialistas de diversas áreas^17^^,^^18^.

A ferramenta AGREE II^11^ destina-se: (1) aos profissionais de saúde, que podem realizar uma avaliação de uma diretriz antes de adotar suas recomendações na prática clínica; (2) aos elaboradores de diretrizes, para que possam seguir uma metodologia de desenvolvimento estruturada e rigorosa; (3) aos gestores de políticas de saúde, com objetivo de auxiliá-los a decidir quais DCs podem ser utilizadas para embasar a tomada de decisões em políticas de saúde; e (4) aos educadores, com o intuito de aprimorar suas habilidades de avaliação crítica e reforçar quais as competências essenciais no desenvolvimento das DCs para que elas possam embasar decisões clínicas. Um total de 33 traduções oficiais da AGREE^10^ (primeira versão) e da AGREE II^11^ estão disponíveis e são utilizadas pela comunidade internacional^11^.

As doenças vasculares extracardíacas apresentam alta prevalência na população mundial com mais de 60 anos de idade. Entre elas, está a doença arterial obstrutiva carotídea extracraniana (DAOC), na qual a grande maioria das intervenções cirúrgicas ocorre em indivíduos totalmente assintomáticos^19^. Considerando que indivíduos assintomáticos têm impacto nulo dessa doença na sua qualidade de vida e que não há intervenção, cirúrgica ou medicamentosa, que não incorra em algum risco (e custo), justifica-se uma avaliação sistemática da qualidade das DCs que norteiam o tratamento dessa doença, visando auxiliar tanto o médico assistente na tomada de decisão quanto o gestor e financiador do sistema de saúde, com o objetivo de avaliar se o recurso disponibilizado está sendo racionalmente utilizado.

A DAOC, caracterizada anatomicamente por uma estenose ou oclusão da artéria carótida, é, majoritariamente, decorrente do processo aterosclerótico que afeta principalmente a bifurcação carotídea, sendo responsável por aproximadamente 20% dos casos de acidente vascular cerebral isquêmico (AVCi)^20^. O tratamento da DAOC consiste na abordagem clínica medicamentosa, com controle clínico rigoroso das doenças associadas (hipertensão arterial sistêmica, diabetes melito, dislipidemia e tabagismo) e na conduta cirúrgica em casos selecionados (endarterectomia carotídea ou angioplastia com implante de *stent*)^19^^,^^20^. Em pacientes sintomáticos, existe consenso quanto ao tratamento cirúrgico para prevenção secundária de um novo AVCi; já nos casos em que os pacientes diagnosticados com DAOC são assintomáticos, a conduta terapêutica cirúrgica para o tratamento da estenose permanece controversa^20^.

O presente estudo consiste em uma avaliação sistemática da qualidade metodológica das DCs que abordam o tratamento da DAOC, por meio da utilização da ferramenta AGREE II.

## MÉTODOS

As buscas foram realizadas em duas bases de dados bibliográficas eletrônicas (PubMed/MEDLINE e SciELO) e no Google Acadêmico. Foram utilizados os seguintes descritores: “carotid artery disease”, “atherosclerotic” e “practice guideline”. Foram incluídos estudos tipo DCs e consensos que abordassem o tratamento da DAOC publicados entre 2000 e 2019. Foram excluídas publicações em língua diversa de inglês, português e espanhol. Dois avaliadores participaram da triagem inicial dos estudos a serem incluídos na seleção das DCs a serem analisadas, por meio da leitura dos títulos e resumos.

Três avaliadores (SGBC, JCR e LPC) avaliaram, independentemente, as DCs incluídas no estudo, pontuando-as desde 1 (baixa qualidade) até 7 (alta qualidade), em cada um dos 23 itens dos seis domínios da ferramenta AGREE II: 1) escopo e finalidade; 2) envolvimento das partes interessadas; 3) rigor do desenvolvimento; 4) clareza e apresentação; 5) aplicabilidade; e 6) independência editorial (Tabela 1). A seguinte fórmula^12^ foi utilizada para a geração do escore ponderado (em percentual) de cada domínio: (escore obtido - escore mínimo possível)/(escore máximo possível - escore mínimo possível) × 100. Todas as avaliações e pontuações foram realizadas de acordo com as orientações do manual de utilização da AGREE II, disponível no *site* do *AGREE Research Trust*^11^.

**Tabela 1:** Itens e domínios do instrumento AGREE II (*Appraisal of Guidelines Research and Evaluation Instrument II*)*.

**Item**	**Conteúdo**	**Domínio**
1	O(s) objetivo(s) geral(is) da(s) diretriz(es) encontra(m)-se especificamente descrito(s).	**Escopo e finalidade**
2	A(s) questão(ões) de saúde coberta(s) pela diretriz encontra(m)-se especificamente descrita(s).	
3	A população (pacientes, público etc.) a quem a diretriz se destina encontra-se especificamente descrita.	
4	A equipe de desenvolvimento da diretriz inclui indivíduos de todos os grupos profissionais relevantes.	**Envolvimento das partes interessadas**
5	Procurou-se conhecer as opiniões e preferências da população-alvo (pacientes, público etc.)	
6	Os usuários-alvo da diretriz estão claramente definidos.	
7	Foram utilizados métodos sistemáticos para a busca de evidências.	**Rigor do desenvolvimento**
8	Os critérios para a seleção de evidências estão claramente descritos.	
9	Os pontos fortes e limitações do corpo de evidências estão claramente descritos.	
10	Os métodos para a formulação das recomendações estão claramente descritos.	
11	Os benefícios, efeitos colaterais e riscos à saúde foram considerados na formulação das recomendações.	
12	Existe uma relação explícita entre as recomendações e as evidências que dão suporte.	
13	A diretriz foi revisada externamente por *experts* antes da sua publicação.	
14	Um procedimento para atualização da diretriz está disponível.	
15	As recomendações são específicas e sem ambiguidade.	**Clareza da apresentação**
16	As diferentes opções de abordagem da condição ou problema de saúde estão claramente apresentadas.	
17	As recomendações-chave são facilmente identificadas.	
18	A diretriz descreve os fatores facilitadores e as barreiras para sua aplicação.	**Aplicabilidade**
19	A diretriz traz aconselhamento e/ou ferramentas sobre como as recomendações podem ser colocadas em prática.	
20	Foram consideradas as potenciais implicações quanto aos recursos decorrentes da aplicação das recomendações.	
21	A diretriz apresenta critérios para o seu monitoramento e/ou auditoria.	
22	O parecer do órgão financiador não exerceu influência sobre o conteúdo da diretriz.	**Independência editorial**
23	Foram registrados e abordados os conflitos de interesse dos membros da equipe que desenvolveram a diretriz.	

* Extraído de AGREE Next Steps Consortium^11^.

Para a análise do grau de concordância entre os avaliadores, foi utilizado o coeficiente de concordância Kappa de Fleiss^21^. Conforme preconizado por Landis & Koch^22^, os valores de Kappa de Fleiss ≥ 0,75 foram considerados representativos de uma concordância excelente entre os avaliadores. Para os domínios nos quais obteve-se valor de Kappa de Fleiss < 0,75, foi preconizada uma reunião dos três avaliadores para discussão e eventual revisão, independente dos escores individuais.

Para a análise da qualidade metodológica global de cada DC, foi utilizada a métrica proposta por Molino et al.^16^, que prioriza a análise do terceiro domínio, por se tratar da avaliação do rigor metodológico no desenvolvimento da DC. Então, para diferenciar a qualidade das DCs, foi utilizado o terceiro domínio para categorizá-las em “alta”, “moderada” ou “baixa” e subcategorizá-las em graus A, B e C, com base na análise dos outros dois domínios de melhor performance na avaliação com a ferramenta AGREE II (Figura 1).

**Figura 1:** Métrica utilizada para avaliação da qualidade metodológica global de cada diretriz clínica (extraída de Molino et al.^16^). AGREE II: *Appraisal of Guidelines Research and Evaluation Instrument II*.

## RESULTADOS

Nove DCs^23^^-^31 foram identificadas e listadas de acordo com o ano de publicação (Tabela 2). Oito DCs foram escritas na língua inglesa, recuperadas da PubMed/MEDLINE, e uma foi escrita em português, recuperada do Google Acadêmico. A maioria das DCs identificadas foi publicada após o ano de 2010 (66%), ou seja, na segunda metade do período estipulado para a busca.

**Tabela 2:** Informações gerais das nove diretrizes clínicas.

**N.º**	**País**	**Título**	**Ano de publicação**
1	Internacional (europeia)	2017 ESC Guidelines on the diagnosis and treatment of peripheral arterial diseases, in collaboration with the European Society for Vascular Surgery.	2017^23^
2	Internacional (europeia)	Management of atherosclerotic carotid and vertebral artery disease: 2017 clinical practice guidelines of the European Society for Vascular Surgery.	2017^24^
3	Brasil	Doença carotídea extracraniana. Diagnóstico e tratamento.	2015^25^
4	Estados Unidos	ASA/ACCF/AHA/AANN/AANS/ACR/ASNR/CNS/SAIP/SCAI/SIR/SNIS/SVM/SVS Guideline on the Management of Patients With Extracranial Carotid and Vertebral Artery Disease: Executive Summary.	2011^26^
5	Estados Unidos	Updated Society for Vascular Surgery guidelines for management of extracranial carotid disease.	2011^27^
6	Reino Unido	Carotid artery stent placement for asymptomatic extracranial carotid stenosis.	2011^28^
7	Estados Unidos	Management of atherosclerotic carotid artery disease: Clinical practice guidelines of the Society for Vascular Surgery.	2008^29^
8	Estados Unidos	Primary prevention of ischemic stroke.	2006^30^
9	Reino Unido	Guidelines on the management of secondary prophylaxis of vascular events in stable patients in primary care.	2004^31^

Os escores iniciais de cada domínio e a avaliação global inicial das nove DCs estão dispostos na Tabela 3. Exceto pelo segundo domínio (envolvimento das partes interessadas), houve concordância excelente entre os avaliadores. Realizou-se, então, uma reunião dos três avaliadores, para discussão e possibilidade de eventual revisão das pontuações atribuídas às DCs no segundo domínio. Após a reunião de discussão, houve total liberdade para que cada avaliador revisse (ou não) individualmente sua pontuação inicial, sendo recalculado o coeficiente de concordância e chegando-se ao resultado final do segundo domínio, agora também com concordância excelente (excelente ou total concordância) entre os avaliadores (Tabela 4).

**Tabela 3:** Escores iniciais dos seis domínios das nove diretrizes clínicas utilizando a ferramenta AGREE II (*Appraisal of Guidelines Research and Evaluation Instrument II*).

**DC (ano)**	**Domínio 1** Escopo e finalidade*	**Domínio 2** Envolvimento das partes interessadas*	**Domínio 3** Rigor do desenvolvimento*	**Domínio 4** Clareza da apresentação*	**Domínio 5** Aplicabilidade*	**Domínio 6** Independência editorial*
**1 (2017)**	100,00	80,00	98,61	100,00	84,72	100,00
**2 (2017)**	100,00	100,00	91,60	100,00	91,60	100,00
**3 (2015)**	72,22	3,70	16,66	78,18	19,44	19,44
**4 (2011)**	98,14	96,29	96,52	100,00	80,00	80,00
**5 (2011)**	77,77	88,88	77,77	100,00	68,05	72,22
**6 (2011)**	100,00	96,29	92,36	98,14	72,22	88,88
**7 (2008)**	100,00	77,77	83,33	100,00	58,33	94,44
**8 (2006)**	83,33	75,92	75,69	92,59	66,66	91,66
**9 (2004)**	98,14	79,62	56,94	96,29	58,33	69,44
Kappa de Fleiss**	0,962	0,715	0,793	0,928	0,889	0,803

DC: diretriz clínica.

* Valores dos escores em percentual;

** Coeficiente de concordância Kappa de Fleiss.

**Tabela 4:** Escores finais (após reunião dos avaliadores) dos seis domínios e avaliação global da qualidade metodológica das nove diretrizes clínicas utilizando a ferramenta AGREE II (*Appraisal of Guidelines Research and Evaluation Instrument II*).

**DC (ano)**	**Domínio 1** Escopo e finalidade*	**Domínio 2** Envolvimento das partes interessadas*	**Domínio 3** Rigor do desenvolvimento*	**Domínio 4** Clareza da apresentação*	**Domínio 5** Aplicabilidade*	**Domínio 6** Independência editorial*	Avaliação global da qualidade metodológica**
**1 (2017)**	100,00	80,00	98,61	100,00	84,72	100,00	ALTA - A
**2 (2017)**	100,00	100,00	91,60	100,00	91,60	100,00	ALTA - A
**3 (2015)**	72,22	1,80	16,66	78,18	19,44	19,44	BAIXA - A
**4 (2011)**	98,14	96,29	96,52	100,00	80,00	80,00	ALTA - A
**5 (2011)**	77,77	88,88	77,77	100,00	68,05	72,22	MODERADA - A
**6 (2011)**	100,00	96,29	92,36	98,14	72,22	88,88	ALTA - A
**7 (2008)**	100,00	77,77	83,33	100,00	58,33	94,44	ALTA - A
**8 (2006)**	83,33	57,40	75,69	92,59	66,66	91,66	MODERADA - A
**9 (2004)**	98,14	74,10	56,94	96,29	58,33	69,44	BAIXA - A
kappa***	0,962	0,813	0,793	0,928	0,889	0,803	-

DC: diretriz clínica.

* Valores dos escores em percentual;

** Avaliação global de qualidade conforme métrica proposta por Molino et al.^16^;

*** Coeficiente de concordância Kappa de Fleiss.

Considerando-se a avaliação global da qualidade metodológica das DCs e utilizando-se a métrica proposta por Molino et al.^16^, cinco diretrizes foram consideradas de alta qualidade (ALTA A), o que corresponde a 55% das DCs disponíveis; duas diretrizes foram consideradas de qualidade moderada (MODERADA A); e duas diretrizes foram consideradas de baixa qualidade (BAIXA A) (Tabela 4).

## DISCUSSÃO

Quanto à avaliação global da qualidade metodológica das DCs, se considerarmos apenas as publicadas em inglês e disponíveis na PubMed/MEDLINE, 62,5% foram consideradas como de alta qualidade (todas ALTA A). Quanto à avaliação negativa da qualidade metodológica global da única DC disponível em português, conforme também encontrado na avaliação de qualidade de DCs sobre doenças cardíacas^32^ e sobre outras doenças crônicas^16^^,^^33^, há bastante espaço para melhoria da qualidade metodológica por meio do uso de instrumentos como o AGREE II na fase de elaboração. Tão importante quanto isso, fica evidente a necessidade de que leitores de DCs e médicos assistenciais recorram, também, a ferramentas de fácil utilização, como a AGREE II, durante a leitura crítica das DCs que utilizam para embasar suas decisões frente a pacientes^32^.

Hoffmann-Eßer et al.^34^, em uma pesquisa *on-line* com 376 pesquisadores, evidenciaram que os domínios que mais influenciam na avaliação da qualidade metodológica global de uma DC são os domínios três (rigor no desenvolvimento) e seis (independência editorial). Em nosso estudo, as duas DCs que foram classificadas como de baixa qualidade também tiveram as piores pontuações nesses dois domínios. Destaca-se, assim, tanto a importância do domínio três, que contém o maior número de itens avaliados (oito itens), quanto a importância do domínio seis, apesar de contemplar apenas dois itens.

Presume-se que o ano de publicação da nona DC (2004), ano subsequente ao ano de publicação da primeira versão do instrumento AGREE^10^, seja um fator relevante para a baixa qualidade metodológica global, visto que o uso da ferramenta AGREE ainda estava no início de sua difusão. Já a terceira DC, apesar de mais recente, pode não ter considerado a ferramenta AGREE II^11^ ou outras existentes^8^^,^^9^ como base para sua elaboração.

Uma das restrições relacionadas à utilização da ferramenta AGREE II é a ampla variabilidade da sua escala (1 a 7) para pontuação de cada um dos 23 itens por cada avaliador, podendo levar a um grau não desprezível de subjetividade por parte de cada avaliador^32^. Entretanto, o elevado grau de concordância inter-avaliadores, por nós encontrado, evidencia que a AGREE II pode superar esse eventual viés. Mesmo no único domínio no qual houve a discussão e repontuação (domínio dois), o grau de concordância inicial já estava bem próximo a excelente (Kappa de Fleiss inicial de 0,715). Isso é consistente com estudos que confirmam a validade da AGREE II como ferramenta de avaliação de DCs em diversas especialidades médicas, mesmo quando há algum grau de heterogeneidade entre os avaliadores^35^.

Algumas críticas à aplicabilidade do instrumento AGREE II em DCs de doenças cirúrgicas são destacadas em uma proposta de protocolo recente^36^, que ressalta a dificuldade em se abordar a custo-efetividade por diferentes motivos: por ser um item muito escasso na literatura cirúrgica, pela variabilidade em experiência cirúrgica em diversos países, pela necessidade de aplicação do instrumento em diferentes ambientes de saúde e pelos comparadores comuns para atender aos detalhes das intervenções mais complexas. Esse protocolo propôs, então, uma extensão ao instrumento AGREE para avaliação de DCs de doenças cirúrgicas, com a inclusão de uma lista de verificação com itens específicos relacionados às peculiaridades das mesmas. Assim, o objetivo do desenvolvimento dessa extensão seria ampliar a aplicabilidade do instrumento e aumentar o seu valor para as DCs de prática clínica em cirurgia.

Uma limitação do presente estudo foi a participação de apenas um subgrupo específico de avaliadores. Os três avaliadores eram médicos especialistas em cirurgia vascular que têm como atividade principal a assistência, não havendo a participação de outros avaliadores, como gestores, educadores e/ou elaboradores de DCs. Também não houve participação de médicos assistentes de outras especialidades que participam na tomada de decisões frente a pacientes portadores de DAC (p. ex., neurologistas e cardiologistas). Outra ressalva deve ser feita quanto ao fato de que o instrumento AGREE II foi desenvolvido para avaliar a qualidade metodológica do desenvolvimento de DCs, não sendo adequado para a análise específica do mérito de conteúdo das mesmas, conforme já pontuado por outros autores que também utilizaram o instrumento para avaliar a qualidade de DCs direcionadas a outras doenças vasculares^37^.

## CONCLUSÕES

Com base em nossos achados, podemos concluir o seguinte: (1) foi factível a utilização do AGREE II para a avaliação de DCs sobre o tratamento da DAC com alto grau de concordância inter-avaliadores; e (2) a maioria das DCs sobre o tratamento da DAC publicadas na língua inglesa tem alta qualidade metodológica global.
